# A multivariate model for the meta-analysis of study level survival data at multiple times

**DOI:** 10.1002/jrsm.1112

**Published:** 2014-02-27

**Authors:** Dan Jackson, Katie Rollins, Patrick Coughlin

**Affiliations:** a Biostatistics Unit, MRCCambridge, UK; b Department of Vascular Surgery, Addenbrookes HospitalCambridge, UK

**Keywords:** Bayesian modelling, critical leg ischemia, multivariate meta-analysis, survival analysis, random effects models

## Abstract

Motivated by our meta-analytic dataset involving survival rates after treatment for critical leg ischemia, we develop and apply a new multivariate model for the meta-analysis of study level survival data at multiple times. Our data set involves 50 studies that provide mortality rates at up to seven time points, which we model simultaneously, and we compare the results to those obtained from standard methodologies. Our method uses exact binomial within-study distributions and enforces the constraints that both the study specific and the overall mortality rates must not decrease over time. We directly model the probabilities of mortality at each time point, which are the quantities of primary clinical interest. We also present *I*^2^ statistics that quantify the impact of the between-study heterogeneity, which is very considerable in our data set. © 2014 The Authors. *Research Synthesis Methods* published by John Wiley & Sons, Ltd.

## 1. Introduction

Meta-analysis, the statistical process of combining the results from separate studies concerned with the same treatment or issue, is now well established. In particular, this is often applied to survival data, where the outcome of interest is death. If interest lies in the probability of death by a particular time point, then a standard univariate meta-analysis using studies that provide the necessary mortality rates, or sufficient information for these to be ascertained, provides valid inferences under the accompanying model's assumptions. When interest lies in the probability of death at several different time points, a simple and common approach is to perform multiple univariate meta-analyses using the available mortality rates at each time. Because the event of interest (death) is binary, outcomes such as the log odds (for non-comparative data) or the log odds ratio (for comparative data) are typically used in these analyses.

Despite its popularity, this repeated use of univariate meta-analysis presents immediate issues. First of all, if different studies provide mortality rates at different time points, for example, then there is no guarantee that the estimated mortality rates will not decrease over time. If this occurs, then the resulting statistical inference immediately loses its face validity. Furthermore, this approach is inefficient because it does not allow the often closely related measures of mortality at different time points to draw strength from each other. For example, a study may not provide the mortality rate at a time close enough to the time of interest, but instead provide the mortality at a slightly earlier or later time. This clearly gives some information about the mortality rate at the time point of interest, and at the very least, it provides a bound or bounds for this, but this is ignored by a univariate meta-analysis. Arends *et al*. (2008) argue that ‘analysing each time point separately and thus, carrying out multiple meta-analyses on the same data set, is inefficient and could lead to inappropriate conclusions’.

Whilst recognising the usefulness of the methodologies that have been developed, our meta-analytic data set (Rollins *et al*., 2013) motivated us to develop a new type of multivariate meta-analysis model. In particular, we decided to enforce the constraint that mortality rates cannot decrease over time, which some methodologies do not demand (e.g. Dear, 1994; Arends *et al*., 2008). We also wanted to avoid directly working with hazard functions (e.g. Fiocco *et al*., 2009; Wang, 2012) because we wanted to instead explicitly model the quantities of interest, which are the probabilities of death following treatment at multiple time points. By modelling the probabilities of death in the way we propose, we avoid having to convert statistical inferences involving hazard functions to estimated probabilities of death for our clinical colleagues to interpret. Finally, we wanted to avoid using normal approximations because the mortality rates at early time points are low.

Our application is perhaps unusual because our question, and so the data collected to answer it, does not involve comparing the effectiveness of different treatment groups. Hence, the methods that have been developed for extracting treatment effect outcome data from survival analysis output from comparative studies (Parmar *et al*., 1998; Williamson *et al*., 2002) are not directly useful here. More specifically, our application concerns patients who require treatment for critical leg ischemia. These patients require lifelong follow-up, and 4-year survival rates of 40% are reported (Santilli and Santilli, 1999). We wished to quantify the average mortality rate of patients following treatment using a meta-analysis. This motivated us to collect data and subsequently develop our methodology. We found that studies give mortality rates at seven distinct time points, all of which were of interest. Only study level data are available to us; but in situations where individual patient data are available, more sophisticated methods of analysis are possible (Tudur-Smith *et al*., 2005; Siannis *et al*., 2010).

Multivariate meta-analysis (van Houwelingen *et al*., 2002; Nam *et al*., 2003; Jackson *et al*., 2011) has been developed in order to allow related outcomes, such as mortality rates at different times, to borrow strength from each other. Our methodology was inspired by this idea but the model that follows in Section 4 is not a standard multivariate meta-analysis model. The rest of the paper is set out as follows. In Section 2, we describe our motivating data set, and in Section 3, we present the results from standard analyses of these data. In Section 4, we present our new model, and in Section 5, we apply it to our data. We discuss some possible extensions in Section 6, and we conclude with a discussion in Section 7.

## 2. Motivating example: critical leg ischemia

A representative selection of the large meta-analytic data set we collected is shown in Table 1; the full data set, and the computing code, is available in the supporting materials that accompany this paper. The data are comprised of 115 mortality rates of patients from 50 studies following some form of treatment for critical leg ischemia. There are 28 517 patients in total, of whom 7907 are observed to die. Studies report the mortality rates at some, but not all, of seven time points: 30 days, 6 months, 1, 2, 3, 4 and 5 years. For example, in Table 1, we see that the first study only reported the mortality rate after 1 year (85/525) but the second reported this at both 30 days (10/171) and at 4 years (118/171). The mortality rates shown in Table 1 are cumulative so that, for example, the 118 deaths that the second study reports at 4 years include the 10 deaths at 30 days. Because we are interested in the average mortality rate following some form of treatment, the study specific mortality rates in Table 1 are across all treatment arms.

**Table 1:** The critical leg ischaemia data. *N* is the number of patients in each study, and the times, j = 1, 2 ···, 7, refer to 30 days, 6 months and 1, 2, 3, 4 and 5 years mortality, respectively. The numbers of deaths at each of these time points are cumulative.

Study (*i*)	*N*	Deaths						
		*j* = 1	*j* = 2	*j* = 3	*j* = 4	*j* = 5	*j* = 6	*j* = 7
1	525			85				
2	171	10					118	
3	208	11	38	60				
.	.	.	.	.	.	.	.	.
.	.	.	.	.	.	.	.	.
50	315	22						129

Although we collected a large meta-analytic data set, obtaining suitable outcome data was difficult because the overall mortality rates at particular times is rarely a main concern in the study reports that comprise the meta-analysis. Instead they are primarily interested in the relative effectiveness of the treatments that they consider, so the overall mortality rates that we require are often partially or not reported. However, where these rates were reported in tables, abstracts or the text they were included in our data set, where any percentages that were quoted were converted to deaths by multiplying by the sample size and rounding to the nearest whole number. We did not attempt to ascertain further mortality rates using values read off from survival curves but, for example, the method proposed by Guyot *et al*. (2012) could be used to augment our data set. We were perhaps fortunate not to have to resort to this strategy, because the mortality rates provided directly in the study reports were sufficient to provide us with a suitable and, in meta-analytic terms, large data set. However in other applications, this may be necessary. By using only the mortality rates reported within studies, we assume that the mortality rates quoted are not given because these rates achieve statistical significance or because of some other statistical criterion that might result in bias.

Modelling the data directly in Table 1 is difficult because the mortality rates from the same study are not independent; because they are cumulative, positive correlations are invoked between mortality rates from the same study. Hence, the data used in analysis when applying the proposed method are shown in Table 2, which displays the data differently. Here, *N* and *D* are the number at risk and the number of deaths since the last recorded mortality rate; *j* = 1, 2 ···, 7 and *k* = 1, 2 ···, 7 indicate the start and end of the time interval that the deaths are known to have occurred, respectively. Take study 2, for example, where there are 10 deaths out of 171 patients at 30 days. These deaths are known to have occurred during the first time period, so the corresponding *j* = 1 and *k* = 1 in Table 2. Following these deaths, the remaining 108 deaths from the 161 who survive known to take place between the second and sixth time periods, so the corresponding *j* = 2 and *k* = 6. Ouwens *et al*. (2010) and Jansen (2011) also display their data in the format used in Table 2.

**Table 2:** The critical leg ischaemia data as used in the analysis. The times, j = 1, 2 ···, 7 and k = 1, 2 ···, 7, denote the start and end of the intervals in which deaths are known to have occurred. The numbers of deaths *D* are known to have occurred in these intervals, and *N* is the number at risk in each interval.

Rate	Study(i)	*j*	*k*	*N*	D
1	1	1	3	525	85
2	2	1	1	171	10
3	2	2	6	161	108
4	3	1	1	208	11
5	3	2	2	197	27
6	3	3	3	170	22
.	.	.	.	.	.
.	.	.	.	.	.
114	50	1	1	315	22
115	50	2	7	293	107

The advantage of displaying the data as in Table 2 is because the deaths *D* in this table may be modelled as independent realizations from binomial distributions with size *N* also from Table 2. Here the binomial probabilities are equal to the probability of death in the interval indicated by *j* and *k*, conditional on surviving up to, but not including, time interval *j*. This independence assumption is justifiable on the grounds that the mortality rates at previous time points tell us nothing about the mortality rates at later times conditional on surviving the earlier time point. This independence assumption underlies the modelling that follows in Section 4.

## 3. Standard meta-analyses of the critical leg ischemia data

Rollins *et al*. (2013) present six univariate random effects meta-analyses, using the data in Table 1, in order to quantify the mortality rate at 30 days; 6 months; 1, 2, 3 and 5 years, using data from studies that provide mortality rates at these time points. A univariate random meta-analysis using the mortality rates at 4 years was not presented because only four studies provide outcome data at this time and fitting random effects models with so few observations is problematic (e.g. Jackson and Bowden, 2009); the fixed effect model was thought, a priori, to be inappropriate because of the variety of treatments and patient mixes. Furthermore, the univariate pooled estimate of mortality at 4 years is greater than the univariate pooled estimate at 5 years, which reinforces this potential problem with the repeated use of univariate meta-analyses described in the introduction.

Specifically, these univariate analyses used the usual normal approximation for the empirical log odds of death as the outcomes in random effects meta-analyses, using the standard method originally suggested by DerSimonian and Laird (1986). A few studies provide mortality rates of zero at 30 days, which were treated as halves in analysis to avoid infinite log odds. The pooled estimates of the average log odds and their confidence intervals were transformed to the probability scale, and the results are shown in Table 3. The *I*^2^ statistics from the univariate meta-analyses on the log odds scale are also shown in Table 3 and show that there is very considerable between-study heterogeneity. The mortality rate rises sharply, and after 5 years, the estimated average probability of mortality is 0.46.

**Table 3:** The results from standard meta-analyses of the critical leg ischaemia data. All analyses were performed on the log-odds scale and the results were converted to the probabilities shown. The point estimates and 95% confidence intervals for the probability of death at each time point are from random effects meta-analyses. These results are displayed as the estimate followed by the 95% confidence intervals, and I^2^ statistics are also shown. Outcome data for mortality at 4 years was not used in analysis because only four studies provide data. Two multivariate meta-analyses were performed because the estimation of the six dimensional meta-analysis failed: the first multivariate meta-analysis makes no use of outcome data at 5 years and the second makes no use of outcome data at 6 months.

	Univariate meta-analyses		Multivariate meta-analyses			
Time point	*P* (death)	*I*^2^ (%)	*P* (death)	*I*^2^ (%)	*P* (death)	*I*^2^ (%)
30 days (*j* = 1)	0.04 (0.03, 0.05)	76	0.04 (0.03 0.05)	88	0.04 (0.03, 0.05)	88
6 months (*j* = 2)	0.14 (0.10, 0.20)	93	0.14 (0.10, 0.19)	96	—	—
1 year (*j* = 3)	0.17 (0.15, 0.20)	95	0.17 (0.14, 0.21)	98	0.17 (0.14, 0.21)	98
2 years (*j* = 4)	0.28 (0.21, 0.37)	97	0.22 (0.17, 0.28)	98	0.20 (0.15, 0.27)	98
3 years (*j* = 5)	0.35 (0.27, 0.44)	97	0.34 (0.28, 0.40)	97	0.36 (0.30, 0.42)	96
4 years (*j* = 6)	—	—	—	—	—	—
5 years (*j* = 7)	0.46 (0.33, 0.60)	99%	—	—	0.47 (0.39, 0.56)	98

These separate univariate analyses only use information from studies at the time point in question and do not allow the reported mortality rates to inform the analyses at different time points. Rollins *et al*. (2013) also used a multivariate random effects meta-analysis, which allows this kind of borrowing of strength (Jackson *et al*., 2011) but this presented problems. This is because the data were too thin for the multivariate random effects model to simultaneously model the outcome data from all six time points analysed univariately. Only one study provides outcome data at both 6 months and 5 years and so estimation of the six dimensional meta-analyses by using Stata's *mvmeta* (StataCorp, College Station, Texas, USA) (White, 2011) failed. Despite this, two multivariate random effects meta-analyses (Table 3) using the multivariate method of DerSimonian and Laird (Jackson *et al*., 2010) of reduced dimension, where outcome data at 6 months and 5 years was not used respectively, allow some borrowing of strength for the later time periods. The first of the multivariate meta-analyses shown in Table 3 was also presented in Rollins *et al*. (2013). The ratio of the standard errors of the pooled log odds of death under the random effects and the fixed effect (no between-study heterogeneity) multivariate models are the *R* statistics for each outcome separately (Jackson *et al*., 2012). These *R* statistics can be converted to *I*^2^ = (*R*^2^ − 1)/*R*^2^ statistics, as also suggested by Jackson *et al*, and expressed as percentages. These *I*^2^ statistics, from the two multivariate meta-analyses on the log odds scale, are also provided in Table 3.

However, by assuming normality, this multivariate model makes no use of the fact that mortality rates cannot decrease over time. Furthermore, although the studies are quite large, at the early time points the event is rare, and the adequacy of any normal approximation for these data is questionable. These limitations motivated the new type of multivariate meta-analysis that follows.

## 4. A new multivariate meta-analysis model

In order to develop the model, the following notation will be used. Let *p*_(*i*,*k*)_ denote the probability of death in time period *k* in study *i given survival up to, but not including, this time period*. For the critical leg ischaemia data set, we have 50 studies and 7 periods, so we define *p*_(*i*,*k*)_ for *i* = 1, 2, ···, 50 and *k* = 1, 2, ···, 7. For example, because *k* = 3 denotes the third period (between 6 months and 1 year), *p*_(1,3)_ is the probability of death after 1 year, given survival to 6 months, in the first study. Let *q*_(*i*,*k*)_ = 1 − *p*_(*i*,*k*)_.

Let *P*_(*i*,*j*,*k*)_ denote the probability of death in either period *j* or *k*, *or sometime in between, that is, the interval j to k*, in study *i*, given survival up to, but not including, period *j*. For example, *P*_(1,3,6)_ is the probability of death after 1, 2, 3 or 4 years given survival to 6 months in the first study. We also have *P*_(*i*,*k*,*k*)_ = *p*_(*i*,*k*)_.

We can relate these two types of probabilities using the formula


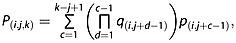
(1)

where any empty products are taken to be one. For example, setting *j* = 3 and *k* = 6, we have


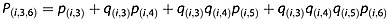


so that the conditional probability of death between periods 3 and 6 is the sum of the conditional probabilities of death at the four time points that comprise this interval. The 115 numbers of deaths that comprise the data in Table 2 are modelled using


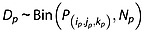
(2)

for *p* = 1, 2, ··· 115, where the subscript *p* denotes which row of Table 2 provides the corresponding variable, and these numbers of deaths *D*_*p*_ are assumed to be independent. This independence assumption is only possible because, for studies that provide numbers of deaths at more than one time point, we model the number of deaths since the mortality rate was previously reported and use conditional probabilities *P*_(*i*,*j*,*k*)_, as explained in when presenting the data in the format shown in Table 2.

An alternative to using equation (2) to describe the data is to model the event of death in all seven periods, and survival beyond 5 years, as mutually exclusive, exhaustive and negatively correlated outcomes using a multinomial distribution (Trikalinos and Olkin, 2008). The correlations implied by this multinomial distribution are accounted for in the structural part of the model, and our proposal has the advantage that non-informative censoring can easily be taken into account, as explained below in Section 6.1.

### 4.1. A random effects model for the parameters of interest

Although modelling the data using equation Section 3 for the unconditional probabilities of death by the time points of interest. These unconditional probabilities are usually of primary clinical interest.

Let *μ*_(*i*,*k*)_ denote the unconditional log odds of death by the *k*th period in study *i*; log odds are defined because a random effects model will be used to describe the heterogeneity using a normal model, and this random effect will be applied on the log-odds scale as in the analyses in Section 3. Because deaths cannot occur before the first period, for *k* = 1, we have that


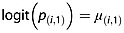
(3)

For *k* ≥ 2, from elementary probability calculations,


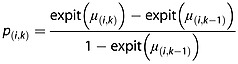
(4)

The heterogeneity will be modelled using the random effect


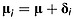
(5)

where **μ**_*i*_ is the vector containing the *μ*_(*i*,*k*)_, **μ** is the vector containing the average unconditional log odds of death by all the time points across studies, and *δ*_*i*_ is the random effect


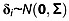
(6)

where *N* denotes the multivariate normal distribution, and **Σ** is the between-study covariance matrix. However by assuming this multivariate random effects distribution, we do not ensure that *μ*_(*i*,*k*)_ is non-decreasing in *k* in equation (4). We therefore suggest replacing equation (4) with


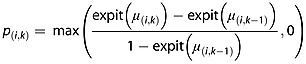
(7)

when modelling the between-study heterogeneity in this way. This means that the probabilities of mortality within-studies are non-decreasing. We impose this constraint on the average probabilities of mortality via the prior specification as explained in the next section.

Together, the former equations fully specify the model: given the **μ**_*i*_ from equations (5) and (6), *p*_(*i*,*k*)_ can be calculated from equations (3) and (7). Then, the *P*_(*i*,*j*,*k*)_ can be calculated using equation (1) and the data modelled using equation (2).

### 4.2. Making inferences

We have seven time points of interest so the model for our data has seven location parameters, the entries of **μ**, and 28 between-study variation parameters, the entries of the symmetrical matrix **Σ**. Inference could be performed using maximum likelihood, but this is computationally challenging because of the high dimension of, and the constraints on, the parameter space. First, **Σ** must be constrained to be positive semi-definite, a challenging task in high dimensions; for example, Stata’s *mvmeta* (White, 2011) uses the Cholesky decomposition to ensure this. Second, because numbers of death must not decrease over time, we must constrain *μ*_*k*_ ≥ *μ*_*j*_ if *k* ≥ *j*, where *μ*_*i*_ is the *i*th entry of **μ**.

In order to overcome these problems, Bayesian analyses, using WinBUGS (Lunn *et al*., 2000) with ‘vague’ priors, were used to approximate likelihood based inferences. However, placing ‘vague’ priors on variance components in meta-analysis is problematic: apparently vague or uninformative priors still have considerable influence for the sample sizes typically encountered in practice (Lambert *et al*., 2005). In order to produce some illustrative results, a ‘vague’ Wishart prior, with rank 7, was used for the inverse of **Σ**, and very flat normal priors, centred at 0 with variance 1000 were used for the *μ*_*i*_, also using WinBUGS' censoring function to ensure that these are non-decreasing in *i*. When specifying this Wishart prior a scale matrix is specified, which represents a prior assessment of the order of magnitude of **Σ**^− 1^. A diagonal matrix with all entries equal to 2 was used for this scale matrix, in order to reflect the considerable heterogeneity anticipated and confirmed in the analyses in Section 3. In application, an assessment of the sensitivity to the prior specification is highly recommended (Jackson *et al*., 2011). Although point estimates of death probabilities were not found to be very sensitive to the scale matrix used for the Wishart prior the intervals are much more so.

This transformation of the log odds of death to probabilities of death was conveniently performed in WinBUGS by defining the additional random variables **θ** = *exp*(**μ**)/(1 + *exp*(**μ**)), where the entries of **θ** denote the parameters of primary interest, the average probabilities of death at each time point.

## 5. Application of the proposed methodology

Two chains were used so that WinBUGS' implementation of the Gelman–Rubin statistic could be used to assess convergence. In the first chain the estimated log-odds from the univariate analyses shown in Table 3 were used as starting values for *μ* (with logit(0.4) used for the sixth entry) and in the second logit(0.1), logit(0.2), …logit(0.7) were used. A burn in of 10 000 iterations was used, and a further 25 000 iterations were performed. With two chains, 50 000 simulated values from the posteriors were used for inference, which required around 12 min on a Windows terminal server to produce. Trace plots were examined, and no trends were apparent. The Gelman–Rubin statistics were stable also indicating that convergence had been reached. The plots of the posterior distributions were unimodal and smooth. The posterior plots for the between-study variances (the diagonal entries of **Σ**) were positively skewed but the posterior plots for the location parameters were much more symmetrical. The posterior means of the probabilities of death **θ** were used as estimates of the seven probabilities of death, and the 2.5 and 97.5% quantiles of the posterior distributions were used to provide the bounds of 95% credible intervals.

In order to quantify the impact of heterogeneity, the model was then fitted in the same way but with **Σ** = 0, which provides a fixed effect model. The ratio of the posterior standard deviations of the entries of **θ** under the random effects (the full model) and fixed effect models give rise to statistics similar in concept to the *R* statistic for each outcome proposed by Jackson *et al*. (2011). These *R* statistics were then converted to *I*^2^ = (*R*^2^ − 1)/*R*^2^ statistics, as also suggested by Jackson *et al*, and expressed as percentages. By using the standard deviations of the posterior probabilities **θ**, rather than log odds ***μ***, we are able to quantify the impact of the heterogeneity directly on the quantities of primary clinical interest. The estimates of **θ**, the average unconditional probabilities of death at the seven time points under the random effects model, are shown in Table 4 with these *I*^2^ statistics.

**Table 4:** The results applying the proposed method to the critical leg ischaemia data. The estimates are the posterior means, which are followed by 95% credible intervals and I^2^ statistics.

Time point	*P* (death)	*I*^2^ (%)
30 days (*j* = 1)	0.03 (0.02, 0.04)	91
6 months (*j* = 2)	0.11 (0.08, 0.15)	93
1 year (*j* = 3)	0.15 (0.12, 0.19)	98
2 years (*j* = 4)	0.22 (0.15, 0.30)	98
3 years (*j* = 5)	0.32 (0.25, 0.40)	99
4 years (*j* = 6)	0.41 (0.31, 0.54)	99
5 years (*j* = 7)	0.47 (0.36, 0.62)	99

The proposed methodology has resulted in similar inferences to the established methods presented in Table 3 but wider intervals are obtained at some time points. Bayesian analyses often provide wider intervals than methods based on frequentist techniques because they allow for parameter uncertainty more fully. Hence, the results in Table 4 better reflect the uncertainty in the estimated probabilities of death whilst providing similar point estimates to the alternative approaches. The proposed approach has also allowed the appropriate estimation of the average mortality rate after 4 years, where this lies between the estimated average mortality rates at 3 and 5 years. This estimation is reasonably precise, but only four studies provide direct evidence about this rate. Hence, the estimation of the average mortality rate at 4 years must rely a great deal upon indirect evidence. The *I*^2^ statistics in Table 4 are even larger than those in Table 3, which makes intuitive sense because the proposed method provides wider intervals. However, the *I*^2^ statistics from the proposed method are based on the probability scale, whereas those from the standard analyses in Section 3 are based on analyses on the log-odds scale. All the various *I*^2^ statistics are very large, as anticipated because of the clinically heterogeneous nature of the studies.

## 6. Extensions of the model

Several extensions of the proposed methodology are immediate. In particular, the model may be extended to include covariates by extending equation (5) to


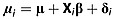


where **X**_*i*_ is the design matrix containing covariates associated with study *i*, and ***β*** is the vector of covariate effect parameters. This is analogous to extending a multivariate meta-analysis to a multivariate meta-regression (White, 2011).

In the context of comparative trials, two approaches for estimating a treatment effect present themselves. First of all, we could include an indicator for the treatment group as a covariate in the formerly proposed extension and use this model for the outcomes in each trial arm, rather than the trial as a whole. This idea has the advantage that it is a simple extension of the proposed methodology; but the drawback is that, by assuming the control group outcomes follow a probability distribution, inter-study information is recovered, which might result in bias (Senn, 2010). Perhaps a better alternative is to use fixed effects for the control group outcomes, or the average study specific outcome, and assume that the true treatment group outcomes differ from the control group by the treatment effect. These treatment effects may be modelled using either fixed or random effects as desired. Other possibilities for extending the methodology for comparative data may also be possible, and this provides a possible avenue for further work.

### 6.1. Censoring

All the analyses performed previously assume no ‘drop out’, that is, that no participants are censored. A few studies provide good information about drop-out rates but most provide partial or no such information (Rollins *et al*., 2013). For consistency, drop out has been ignored in our analyses, and so, particularly at later time points, the estimated probabilities of death are biased downwards. Although follow-up is generally very good in this context, because patients with critical leg ischemia require lifelong follow-up, in other contexts this may be a more serious cause for concern.

The methodology is easily extended to deal with drop-out rates where these are known and where any censoring is assumed to non-informative: the sample size values of *N* may simply be reduced accordingly. In situations were high drop-out rates are expected, but not reported, sensitivity analyses could be performed assuming particular reductions of *N*. For example, constant rates of censoring in studies could be assumed so that all *N* are reduced by quantities that are determined by the assumed rate of censoring and the length of the time intervals. However, this type of sensitivity analysis requires information regarding the extent of censoring from the study reports, or perhaps expert subject knowledge about the likely extent of this. By applying the method over short periods of follow-up, concerns regarding unknown loss to follow-up can be reduced but not necessarily completely avoided. Censoring therefore raises very real concerns about bias in practice; but with appropriate information, the methodology can directly take this into account.

## 7. Discussion

We have developed some novel methodology for the meta-analysis of study level survival data. The model is a type of random effects multivariate meta-analysis model where the structure imposes that mortality rates, both within studies and on average, must not decrease over time. The proposed method avoids the use of hazard functions and uses the probabilities of death directly. It therefore leads to the type of inference we desired in a more immediate way.

Data in the form presented in Table 2 are also modelled using equation (2) by Ouwens *et al*. (2010); Jansen (2011) and Jansen and Cope (2012) in the context of network meta-analysis. These authors use parametric models, so their use of hazard functions to describe their models is both natural and standard. However, this means that the probabilities of death in each time interval must be calculated from the hazard function, and so are dependent on the modelling assumptions. We instead adopted a distribution free approach within studies and took the opportunity to work with the probabilities of death in each time interval directly. In situations where a parametric approach is preferred, analyses based on the hazard function are more likely to be adopted. Parmar *et al*. (1998) suggest a simple method for estimating an overall survival curve from published survival curves in their Section 7. Because of its simplicity, this type of approach might be considered preferable when a crude overall survival curve is desired, but we are confident that our approach is more appropriate when formal inference about time specific survival probabilities are required.

A Bayesian version of the methodology was presented, but this was merely to take advantage of the Markov Chain Monte Carlo algorithms that have been developed and hence, for computational convenience. Because the sample size is reasonably large, we anticipate that our results in Section 5 will be similar to those from fitting our model using a maximum likelihood. In order to avoid the problems associated with prior specification, an entirely likelihood-based approach for fitting the model may form the subject of future work, but here WinBUGS was used because it provides a flexible and convenient framework for fitting non-standard models of this kind.

If there were very many time points of interest, then the proposed approach loses its appeal because the resulting multivariate model would be of high dimension. Hence, the decisions concerning the number of time periods used, and therefore their lengths, may be influenced by the data available. The amount of data needed to reliably fit multivariate meta-analysis models is an open issue, but if the data are too thin, then imprecise estimation is almost inevitable. Our method is therefore completely impractical in situations where studies give mortality data at completely different time points. However, the ambitions of most models used in meta-analysis are fairly modest, and we feel that we have developed a useful framework for the meta-analysis of study level survival data. The advantages of our approach are that we avoid using normal approximations, we enforce the condition that mortality probabilities cannot decrease over time, and *I*^2^ statistics are readily available. The methodology was entirely motivated by our application and extensions, such as those described in Section 8, are of interest, and we hope that others will be motivated to take our ideas forwards.
