# Supplementary material for: A multivariate model for the meta-analysis of study level survival data at multiple times
Source: Res Synth Methods. 2014 Feb 27;5(3):264–72. doi: 10.1002/jrsm.1112 (PMC4433770; doi:10.1002/jrsm.1112)
Supplement: Supplementary file 1 [file jrsm0005-0264-sd1.doc]

The model; “n” is the number of mortality rates (115) and “studies” is the number of studies (50).

model{

for(i in 1:n)

{

# Calculate probabilities:

P[i, 1, 1] <- p[i,1]

P[i, 1, 2] <- P[i, 1, 1] + (1-p[i,1]) * p[i,2]

P[i, 1, 3] <- P[i, 1, 2] + (1-p[i,1]) * (1-p[i,2]) * p[i,3]

P[i, 1, 4] <- P[i, 1, 3] + (1-p[i,1]) * (1-p[i,2]) * (1-p[i,3]) * p[i,4]

P[i, 1, 5] <- P[i, 1, 4] + (1-p[i,1]) * (1-p[i,2]) * (1-p[i,3]) * (1-p[i,4]) * p[i,5]

P[i, 1, 6] <- P[i, 1, 5] + (1-p[i,1]) * (1-p[i,2]) * (1-p[i,3]) * (1-p[i,4]) * (1-p[i,5]) * p[i,6]

P[i, 1, 7] <- P[i, 1, 6] + (1-p[i,1]) * (1-p[i,2]) * (1-p[i,3]) * (1-p[i,4]) * (1-p[i,5]) * (1-p[i,6]) * p[i,7]

P[i, 2, 2] <- p[i,2]

P[i, 2, 3] <- P[i, 2, 2] + (1-p[i,2]) * p[i,3]

P[i, 2, 4] <- P[i, 2, 3] + (1-p[i,2]) * (1-p[i,3]) * p[i,4]

P[i, 2, 5] <- P[i, 2, 4] + (1-p[i,2]) * (1-p[i,3]) * (1-p[i,4]) * p[i,5]

P[i, 2, 6] <- P[i, 2, 5] + (1-p[i,2]) * (1-p[i,3]) * (1-p[i,4]) * (1-p[i,5]) * p[i,6]

P[i, 2, 7] <- P[i, 2, 6] + (1-p[i,2]) * (1-p[i,3]) * (1-p[i,4]) * (1-p[i,5]) * (1-p[i,6]) * p[i,7]

P[i, 3, 3] <- p[i,3]

P[i, 3, 4] <- P[i, 3, 3] + (1-p[i,3]) * p[i,4]

P[i, 3, 5] <- P[i, 3, 4] + (1-p[i,3]) * (1-p[i,4]) * p[i,5]

P[i, 3, 6] <- P[i, 3, 5] + (1-p[i,3]) * (1-p[i,4]) * (1-p[i,5]) * p[i,6]

P[i, 3, 7] <- P[i, 3, 6] + (1-p[i,3]) * (1-p[i,4]) * (1-p[i,5]) * (1-p[i,6]) * p[i,7]

P[i, 4, 4] <- p[i,4]

P[i, 4, 5] <- P[i, 4, 4] + (1-p[i,4]) * p[i,5]

P[i, 4, 6] <- P[i, 4, 5] + (1-p[i,4]) * (1-p[i,5]) * p[i,6]

P[i, 4, 7] <- P[i, 4, 6] + (1-p[i,4]) * (1-p[i,5]) * (1-p[i,6]) * p[i,7]

P[i, 5, 5] <- p[i,5]

P[i, 5, 6] <- P[i, 5, 5] + (1-p[i,5]) * p[i,6]

P[i, 5, 7] <- P[i, 5, 6] + (1-p[i,5]) * (1-p[i,6]) * p[i,7]

P[i, 6, 6] <- p[i,6]

P[i, 6, 7] <- P[i, 6, 6] + (1-p[i,6]) * p[i,7]

P[i, 7,7]<-p[i,7]

logit(p[i,1])<-mu[1] + delta[study[i],1]

for(k in 2:7)

{

p[i,k]<-max((exp(mu[k]+delta[study[i],k])*(1+exp(mu[k-1]+delta[study[i],(k-1)]))-exp(mu[k-1]+delta[study[i],(k-1)])*(1+exp(mu[k]+delta[study[i],k])))/(1+exp(mu[k]+delta[study[i],k])),0)

}

#Model outcomes; start and end are the start and end of the intervals the deaths occur in (denoted by j and k in the paper)

probs[i]<-P[study[i], start[i], end[i]]

D[i]~dbin(probs[i], N[i])

}

# Generate random effects:

for(i in 1:studies)

{

delta[i, 1:7]~ dmnorm(zero[] , Omega[,])

}

#Convert odds to probabilities. theta[1:7] are the parameters of primary interest.

for(i in 1:7)

{

theta[i]<-exp(mu[i])/(1+exp(mu[i]))

}

# Priors

mu[1]~dnorm(0,0.001) I(, mu[2])

mu[2]~dnorm(0,0.001) I(mu[1], mu[3])

mu[3]~dnorm(0,0.001) I(mu[2], mu[4])

mu[4]~dnorm(0,0.001) I(mu[3], mu[5])

mu[5]~dnorm(0,0.001) I(mu[4], mu[6])

mu[6]~dnorm(0,0.001) I(mu[5], mu[7])

mu[7]~dnorm(0,0.001) I(mu[6], )

Omega[1 : 7 , 1 : 7] ~ dwish(R[ , ], 7)

Sigma[1 : 7 , 1 : 7] <- inverse(Omega[ , ])

}

The data:

list(studies=50, D=c(85, 10, 108, 11, 27, 22, 87, 52, 220, 103, 61, 45, 32, 89,

70, 291, 245, 155, 62, 14, 17, 83, 3, 20, 32, 0, 18, 0, 28, 0,

40, 2, 31, 0, 5, 3, 2, 31, 4, 268, 80, 1, 1, 10, 3, 109, 41,

864, 26, 74, 2, 5, 13, 113, 41, 13, 38, 38, 187, 5, 6, 8, 1,

15, 6, 24, 33, 1, 118, 1288, 8, 7, 11, 51, 37, 39, 13, 65, 0,

28, 31, 15, 6, 14, 20, 26, 22, 2, 20, 8, 17, 33, 15, 209, 89,

44, 23, 11, 11, 261, 873, 8, 9, 10, 13, 7, 9, 22, 12, 21, 39,

8, 71, 22, 107), N=c(525, 171, 161, 208, 197, 170, 1039, 584, 532, 312, 209, 148,

103, 278, 1425, 1355, 1064, 819, 149, 106, 92, 510, 101, 98,

78, 197, 197, 106, 106, 155, 155, 103, 101, 108, 108, 103, 103,

101, 554, 550, 241, 140, 139, 138, 128, 259, 150, 1840, 376,

350, 137, 135, 314, 301, 443, 402, 229, 1404, 1366, 180, 175,

113, 105, 145, 108, 102, 78, 993, 992, 5787, 220, 212, 205, 194,

143, 134, 526, 513, 100, 100, 72, 41, 169, 163, 149, 129, 103,

235, 233, 213, 205, 188, 155, 1560, 1351, 120, 108, 85, 74, 4929,

4668, 109, 101, 92, 82, 69, 103, 94, 213, 201, 180, 395, 387,

315, 293), start=c(1, 1, 2, 1, 2, 3, 1, 1, 2, 4, 5, 6, 7, 1, 1, 2, 4, 6, 1, 1,

4, 1, 1, 2, 5, 1, 2, 1, 2, 1, 2, 1, 2, 1, 2, 3, 1, 2, 1, 2, 1,

1, 2, 4, 6, 1, 1, 1, 1, 2, 1, 2, 1, 2, 1, 3, 1, 1, 2, 1, 2, 1,

2, 1, 1, 2, 5, 1, 2, 1, 1, 2, 3, 4, 6, 1, 1, 2, 1, 2, 4, 6, 1,

2, 4, 5, 6, 1, 2, 4, 5, 6, 7, 1, 3, 1, 1, 3, 5, 1, 2, 1, 2, 3,

4, 5, 1, 2, 1, 2, 4, 1, 2, 1, 2), end=c(3, 1, 6, 1, 2, 3, 3, 1, 3, 4, 5, 6, 7, 4, 1, 3, 5, 7, 2, 3,

5, 4, 1, 4, 7, 1, 3, 1, 5, 1, 4, 1, 5, 1, 2, 3, 1, 5, 1, 7, 7,

1, 3, 5, 7, 5, 3, 4, 1, 3, 1, 2, 1, 4, 2, 3, 3, 1, 3, 1, 4, 1,

3, 2, 1, 4, 7, 1, 7, 3, 1, 2, 3, 5, 7, 2, 1, 3, 1, 3, 5, 7, 1,

3, 4, 5, 6, 1, 3, 4, 5, 6, 7, 2, 3, 4, 2, 4, 5, 1, 3, 1, 2, 3,

4, 5, 1, 3, 1, 3, 5, 1, 5, 1, 7), n=115, study=c(1, 2, 2, 3, 3, 3, 4, 5, 5, 5, 5, 5, 5, 6, 7, 7, 7, 7, 8, 9,

9, 10, 11, 11, 11, 12, 12, 13, 13, 14, 14, 15, 15, 16, 16, 16,

17, 17, 18, 18, 19, 20, 20, 20, 20, 21, 22, 23, 24, 24, 25, 25,

26, 26, 27, 27, 28, 29, 29, 30, 30, 31, 31, 32, 33, 33, 33, 34,

34, 35, 36, 36, 36, 36, 36, 37, 38, 38, 39, 39, 39, 39, 40, 40,

40, 40, 40, 41, 41, 41, 41, 41, 41, 42, 42, 43, 44, 44, 44, 45,

45, 46, 46, 46, 46, 46, 47, 47, 48, 48, 48, 49, 49, 50, 50), zero = c(0,0,0,0,0,0,0) , R = structure(

.Data = c(2, 0, 0, 0, 0,0,0, 0, 2, 0, 0, 0,0,0, 0, 0, 2, 0, 0,0,0, 0, 0, 0, 2, 0,0,0, 0, 0, 0, 0, 2,0,0, 0, 0, 0, 0, 0,2,0, 0, 0, 0, 0, 0,0,2), . Dim = c(7, 7)))

Initial values:

list(delta=structure(.Data=c(0, 0, 0, 0, 0, 0, 0, 0, 0, 0, 0, 0, 0, 0, 0, 0, 0, 0, 0, 0,

0, 0, 0, 0, 0, 0, 0, 0, 0, 0, 0, 0, 0, 0, 0, 0, 0, 0, 0, 0, 0,

0, 0, 0, 0, 0, 0, 0, 0, 0, 0, 0, 0, 0, 0, 0, 0, 0, 0, 0, 0, 0,

0, 0, 0, 0, 0, 0, 0, 0, 0, 0, 0, 0, 0, 0, 0, 0, 0, 0, 0, 0, 0,

0, 0, 0, 0, 0, 0, 0, 0, 0, 0, 0, 0, 0, 0, 0, 0, 0, 0, 0, 0, 0,

0, 0, 0, 0, 0, 0, 0, 0, 0, 0, 0, 0, 0, 0, 0, 0, 0, 0, 0, 0, 0,

0, 0, 0, 0, 0, 0, 0, 0, 0, 0, 0, 0, 0, 0, 0, 0, 0, 0, 0, 0, 0,

0, 0, 0, 0, 0, 0, 0, 0, 0, 0, 0, 0, 0, 0, 0, 0, 0, 0, 0, 0, 0,

0, 0, 0, 0, 0, 0, 0, 0, 0, 0, 0, 0, 0, 0, 0, 0, 0, 0, 0, 0, 0,

0, 0, 0, 0, 0, 0, 0, 0, 0, 0, 0, 0, 0, 0, 0, 0, 0, 0, 0, 0, 0,

0, 0, 0, 0, 0, 0, 0, 0, 0, 0, 0, 0, 0, 0, 0, 0, 0, 0, 0, 0, 0,

0, 0, 0, 0, 0, 0, 0, 0, 0, 0, 0, 0, 0, 0, 0, 0, 0, 0, 0, 0, 0,

0, 0, 0, 0, 0, 0, 0, 0, 0, 0, 0, 0, 0, 0, 0, 0, 0, 0, 0, 0, 0,

0, 0, 0, 0, 0, 0, 0, 0, 0, 0, 0, 0, 0, 0, 0, 0, 0, 0, 0, 0, 0,

0, 0, 0, 0, 0, 0, 0, 0, 0, 0, 0, 0, 0, 0, 0, 0, 0, 0, 0, 0, 0,

0, 0, 0, 0, 0, 0, 0, 0, 0, 0, 0, 0, 0, 0, 0, 0, 0, 0, 0, 0, 0,

0, 0, 0, 0, 0, 0, 0, 0, 0, 0, 0, 0, 0, 0, 0), .Dim = c(50, 7)))

Initial values for chain 1:

list(mu=c(-3.18, -1.82, -1.59, -0.94, -0.62, -0.41, -0.16))

Initial values for chain 2:

list(mu=c(-2.2, -1.39, -0.85, -0.41, 0, 0.41, 0.85))
